# Prediction of angiogenesis suppression by myricetin from *Aeginetia indica* via inhibiting VEGFR2 signaling pathway using computer-aided analysis

**DOI:** 10.1016/j.heliyon.2025.e41749

**Published:** 2025-01-07

**Authors:** Marjanur Rahman Bhuiyan, Khondoker Shahin Ahmed, Md Sharif Reza, Hemayet Hossain, Syed Mumtahin Mannan Siam, Shahriar Nayan, Sarah Jafrin, Sadikur Rahman Shuvo, A.F.M.Shahid Ud Daula

**Affiliations:** a Department of Pharmacy, Noakhali Science and Technology University, Sonapur, Noakhali, 3814, Bangladesh; b Chemical Research Division, Bangladesh Council of Scientific and Industrial Research (BCSIR), Dhaka, 1205, Bangladesh; c Institute of Food Science and Technology (IFST), Bangladesh Council of Scientific and Industrial Research (BCSIR), Dhaka, 1205, Bangladesh; d Department of Microbiology, Noakhali Science and Technology University, Sonapur, Noakhali, Bangladesh

**Keywords:** *Aeginetia indica*, VEGFR2, HPLC analysis, Myricetin, Renal cancer

## Abstract

Vascular endothelial growth factor receptor-2 (VEGFR2) plays a pivotal role in promoting angiogenesis and contributing to the growth and progression of renal cancer. Hence, the current investigation was undertaken with the aim of identifying safe and potent phytochemicals from *Aeginetia indica* whole plant extract (AiWE) that can efficiently suppress the overexpression of VEGFR2. HPLC analysis identified and quantified 11 polyphenols in considerable amounts in AiWE. All the compounds showed good binding energies with VEGFR2 in the molecular docking study, except catechin hydrate and rutin hydrate. However, among the polyphenols, myricetin exhibited an almost similar hydrogen bonding pattern with the active site of VEGFR2. The all-atom molecular dynamic simulation revealed that myricetin showed a very stable interaction with the active site of VEGFR2 throughout the simulation. Based on these results, it is suggested that myricetin may inhibit angiogenesis by suppressing the VEGFR2 signaling, thereby impeding the growth and progression of renal cancer.

## 1 Introduction

Cancer is one of the major global health problems associated with uncontrolled cellular growth, proliferation, and metastasis. It is the leading cause of death today, and the number of patients is increasing every year. Scientists have identified about 3000 species of plants containing cytotoxic properties [1]. Plant derived vinca alkaloids, taxol, podophyllotoxins, polyphenols, flavonoids, and catechins are associated with potent anti-tumor activities related to anticancer therapy [2]. The latest cheminformatics and bioinformatics tools provide fields for selecting potential anticancer drug targets. Among the diverse phytochemicals of a plant, it is difficult to identify the actual biologically active anticancer compound. In silico approaches with multiple targeting strategies is the most suitable method to explore the unknown compounds of a plant that might have anticancer properties. A number of target macromolecules and biologically active anticancer compounds have been identified through the years using the in-silico approaches [3]. VEGFR2 (vascular endothelial growth factor receptor-2) is one of the most crucial targets for anticancer treatment approaches. This tyrosine kinase receptor is potentially associated with angiogenesis that promotes metastasis of the tumor cells [4]. Overexpression of VEGFR2 is mostly associated with highly vascularized tumors such as renal cell carcinoma (RCC) [5]. RCC is one of the most common malignancies with solid tumors in one or both kidneys. Inhibition of the overexpression of VEGFR2 can be an evolutionary approach in the path of anti-renal cancer treatment [6].

Catalytic domain of VEGFR2 is bilobed and it acts as a binding site of ATP for auto phosphorylation by conformational changes of these two lobes [7]. The active site is mainly divided into two hydrophobic regions and one linker region. The inhibitors for VEGFR2 are generally divided into two main classes, type I and type II based on the regions of catalytic site where the inhibitors bind. There is a conserved DFG motif (Asp-Phe-Gly) which exerts the variation between type I and type II inhibitors [8]. Based on the position of Phe 1047, there are two conformations of VEGFR2, DFG-in (active) and DFG-out (inactive) [9]. Type I inhibitors specifically interact with DFG-in conformation rendering competition with ATP and forms hydrogen bonds with Cys 919, Glu 917 residues. Type II shows interactions for DFG-out conformation and binds with amino acid residues in the linker region namely Glu 885 and Asp 1046 [10]. Additionally, the crystallographic structures reveal the incompatibility of ATP to the active site in DFG-out conformation causing a decrease of affinity up to seven folds [11]. Considering the phenomenon, finding potential inhibitors to work in both active and inactive conformations of VEGFR2 would be an effective therapeutic approach.

*Aeginetia indica* L. is a holoparasitic plant of the Orobanchaceae family, grows on bamboo and pampas grasses root (Supplementary Fig. 1), known as Nanbangiseru (Japanese) and Guan-Jen-Huang (Chinese). In Bangladesh, *A. indica* is known as “Agienata” and is commonly called “Buishakphul gulu” in the tribal culture. This plant is abundantly found in Japan, China, India, Bangladesh, Taiwan, and other Southeast Asian countries [12]. Traditionally people use *A. indica* as a folk medicine to treat cough, fever, chronic liver disease, diabetes, and arthritis [13,14]. It is also used as a tonic and anti-inflammatory product and applied on wounds for healing [15]. Some previous studies found that the seed extract of *A. indica* contained anti-tumor activities. Seed extracts showed synergistic effects with 5-Fluorouracil at high concentration in inhibiting renal cancer cell growth and metastasis [13]. Recent studies reported that *A. indica* had antiviral activity against hepatitis C virus life cycle [16].

Our previous study evaluated the antidiabetic and hepatoprotective potential of *A. indica* methanol extract and isolated compounds [17]. The present study was designed to investigate the phytochemicals profile, and anti-cancer properties of *A. indica*, considering this plant as an essential source of traditional medicinal products. The crude methanol extract was also subjected to high-performance liquid chromatography (HPLC) to identify the phenolic phytochemicals. In addition, in silico molecular docking and dynamic simulation studies were carried out to identify the compounds responsible for anti-metastatic activity in renal cancer.

## 2 Materials and methods

### 2.1 Plant collection and extract preparation

The parasitic plant *Aeginetia indica* was collected from the Chittagong Hill Tracts in Bangladesh and verified by Khandakar Kamrul Islam, Senior Scientific Officer at the Bangladesh National Herbarium in Dhaka (DACB No. 46478). Following a thorough washing, the plant components underwent a process of shade drying for a duration of one week. Subsequently, they were fragmented into smaller bits and ultimately transformed into a finely powdered form using pulverization. A powdered sample weighing 100 g was subjected to extraction using a solvent composed of 80 % methanol (1 % w/v). After 14 days. the mixture was filtered through Whatman filter paper. The solvent was then subjected to evaporation by means of a rotary evaporator to get the crude *Aeginetia indica* methanol extract (AiME, 6.1 %). Total 6.1 g of yellowish-brown AiME was stored at −4 °C until use to prevent microbial contamination.

### 2.2 HPLC-DAD profiling of phenolic compounds

#### 2.2.1 Sample preparation

Phenolic compounds purchased from Sigma-Aldrich, Germany (Supplementary Table 1) were solubilized in methanol within a 25 mL volumetric flask to create stock standard solutions. The concentrations of stock solutions varied from 4.0 to 50 μg/ml. The requisite volumes of each stock solution were combined and subsequently diluted serially to formulate the working standard solutions. All experimental solutions, including the spiked solution, the AiWE, and the standards, were filtered through a 0.45 μm Nylon 6,6 membrane filter and subsequently degassed under vacuum prior to HPLC analysis. The samples were maintained in darkness at 4 °C within the autosampler unit's rack until the analysis commenced.

#### 2.2.2 HPLC-DAD analysis

*Aeginetia indica* methanol extract (AiME) was subjected to chromatographic analysis to identify and quantify the phenolic profile. High-performance liquid chromatography analysis was conducted on an LC-20 series HPLC system (Shimadzu, Japan) coupled with a degasser, an auto-sampler, a binary solvent delivery unit, a column oven, and a photodiode array detector. In this experiment, Luna C18 (5 μm) reverse phase HPLC column (4.6 × 250 nm) was used as a stationary phase, which was controlled at a fixed 33 °C temperature using an LC solution software system (Shimadzu, Japan). The solution of 1 % acetic acid in acetonitrile (A) and 1 % acetic acid in water (B) composed the mobile phase. The separation was carried out by injecting a 20 μL sample with a flow rate of 0.5 mL/min and setting the detection wavelength at 270 nm. The gradient elution program was introduced as follows: 5–25 % solution A for 0–20 min, 25–40 % solution A for next 21–30 min, 40–60 % solution A for 31–35 min, 60-30 % solution A for 36–40 min, 30-5% solution A for 41–45 min and 5 % solution A for last 46–50 min. All the experimental solutions were filtered through a 0.45 μm Nylon 6, 6 membrane filter (India), and degassed under vacuum. A standard stock solution of phenolic compounds dissolved in methanol containing catechin hydrate (50 μg/ml); gallic acid (20 μg/ml); catechol, (−) epicatechin, and rosmarinic acid (30 μg/ml each); 3,4-dihydroxy benzoic acid (15 μg/ml); vanillic acid, p-coumaric acid, rutin hydrate, caffeic acid, syringic acid, quercetin and trans-ferulic acid (10 μg/ml each); kaempferol and myricetin (8 μg/ml) and trans-cinnamic acid (4 μg/ml), was used for the calibration curve preparation. After the experiment, all acquired data, calibration, and peak integration (Supplementary Tables 2 and 3) were determined using LabSolution software. The retention times and UV absorption spectrum acquired for the test sample were compared with the standard using the calibration curve, which revealed the phenolic profile of the sample. The quantitative value of identified compounds was expressed as mg per 100 g of dry extract.

### 2.3 In silico analysis

#### 2.3.1 Molecular docking

VEGFR2, is a prominent regulator of angiogenesis of certain carcinomas and lymphoma. Consequently, A crystal structure of VEGFR (PDB:4AGD) that are complexed with sunitinib was downloaded in PDB format from the RCSB protein data bank (RCSB PDB) (https://www.rcsb.org/) with specific resolution of proteins 2.81 Å. Afterwards, bonded heteroatoms, water molecules and hydrogens from protein structure were removed by using Pymol version 2.5.2. Swiss-PDB viewer software packages (version 4.1.0) used for optimizing the cleaned protein structure while ensuring the least energy. A list of 40 chemical compounds that are present in plant extract identified by HPLC analysis and isolated in previous research were screened by Lipinski violation rule. Then, nine compounds are shortlisted and considered to be as our ligand for the in-silico study and assumed as the potential inhibitor against of VEGFR2 protein. 3D Structure of 9 ligands were downloaded from online database (https://pubchem.ncbi.nlm.nih.gov/). Moreover, sunitinib has been considered an extra ligand in set for validating the overall docking procedure as it crystalized with the protein structure. Autodoc 4.5. tool used for this docking technique to find their potential binding energies. VEGFR2 protein (PDB: 4AGD) are docked against our 10 ligands (including reference drug sunitinib). After cleaning, the Downloaded protein in PyMol and optimized in SWISS PDB viwer loaded in AutoDoc and then delete the subsequent water molecules. Each of the individual ligands are docked with the gridbox following: (center at X: 44.73, Y: −3.713, Z: −12.605 and dimensions were X: 15.791, Y: 16.694, Z: 16.233)

#### 2.3.2 Molecular dynamics simulation

To investigate the VEGFR2 inhibition potential of the phytocompounds detected in *Aeginetia indica*, molecular dynamics simulation was conducted. Based on molecular docking study, the abundant and most potential phytocompound occupying active and allosteric site of VEGFR2 was selected for molecular dynamics simulation. Both the active and inactive conformation of VEGFR2 (PDB ID: 3B8R and 3B8Q) were used as the receptor protein for dynamics simulation. GROMACS v2021.4 simulation tool was used to meet up the purpose. Receptor protein topology was built by using CHARMM36 force field for GROMACS [18]. Ligand topology was generated through CHARMM general force field (CGenFF) server. Protein and ligand topologies were combined to build the complex. TIP3P water model was applied in the system box containing the protein and ligand. Ions were added to counter balance the whole system. Energy minimization was conducted using 1500 steepest descent minimization steps. Position restraint topology was created to apply restraints to the ligands. The receptor protein and ligand were grouped for temperature coupling. The entire system was equilibrated in NVT and NPT. Finally, molecular dynamics simulation was performed for 25ns. The trajectory file which was generated during the simulation was used for calculating RMSD, RMSF, radius of gyration, number of hydrogen bonds etc.

## 3 Results

### 3.1 HPLC profiling of phenolic compounds

**Fig. 1:**
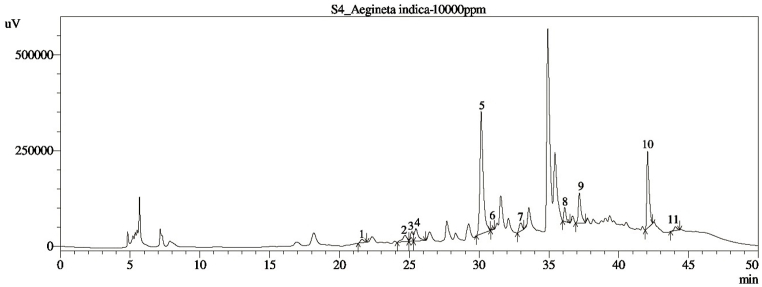
HPLC Chromatogram for methanol extract of *Aegineta indica* (AiME).

The most significant plant-based bioactive compounds that decrease the risk of oxidative stress-related diseases like inflammation, diabetes, and cancer are phenolic compounds. The major phenolic compounds identified and quantified from the HPLC profiling of the *Aeginetia indica* methanol extract are depicted in Table 1. Eleven compounds were matched with the retention time of standard stock solution in the chromatographic analysis (Fig. 1). Among them rutin hydrate (340.62 mg/100 g), and quercetin (143.54 mg/100 g) were identified as the most abundant in *A. indica* extract. Myricetin (105.47 mg/100 g), (−) epicatechin (97.75 mg/100 g), rosmarinic acid (90.73 mg/100 g), catechin hydrate (89.18 mg/100 g), vanillic acid (73.60 mg/100 g), caffeic acid (63.07 mg/100 g) and trans-ferulic acid (59.11 mg/100 g) were also detected at a significantly higher amount whereas trans-cinnamic acid (5.58 mg/100 g) and p-coumaric acid (1.64 mg/100 g) were found at the lowest amount.

**Table 1:** Compounds identified in the methanol extract of *Aegineta indica* (AiME) via HPLC.

Compounds	Amount in AiME (mg/100 g dry extract)	Structure
Rutin hydrate	340.62 ± 0.85	
Quercetin	143.54 ± 0.68	
Myricetin	105.47 ± 0.53	
(−) Epicatechin	97.75 ± 0.51	
Rosmarinic acid	90.73 ± 0.47	
Catechin hydrate	89.18 ± 0.37	
Vanillic acid	73.60 ± 0.33	
Caffeic acid	63.07 ± 0.30	
Trans-Ferulic acid	59.11 ± 0.28	
Trans-Cinnamic acid	5.58 ± 0.17	
p-Coumaric acid	1.64 ± 0.11

### 3.2 In silico analysis

#### 3.2.1 Molecular docking analysis

Molecular docking of phytocompounds (phenolic compounds and previously isolated compounds) and standard sunitinib has been performed with the receptor protein VEGFR2. The binding energies and interactions of phytoconstituents with VEGF2 found in the docking study have been shown in Table 2 and Supplementary Table 4. Sunitinib shows its potentiality by hydrogen binding on CYS919, ASP1046, VAL899 with a high docking score is −8.1. Among the total 27 compounds, 5 compounds showed higher binding scores than the standard VEGFR2 inhibitor sunitinib. These potential compounds are apigenin, quercetin, myricetin, rosmarinic acid, and epicatechin and their binding scores are −9.3, −8.7, −8.5, −8.3, −8.2, respectively. In addition to greater binding energies, myricetin also showed a similar hydrogen bonding pattern to that of sunitinib.

**Table 2:** Binding affinity found after molecular docking.

Ligand	Binding energies (kcal/mol)
Apigenin	−9.3
Quercetin	−8.7
Myricitin	−8.5
Rosmarinic acid	−8.3
Epicatechin	−8.2
Sunitinib	−8.1
Oleic acid	−7.1
p-Coumaric acid	−7.1
Trans-Cinnamic acid	−6.9
Caffeic acid	−6.8
Trans-Ferulic acid	−6.8
Ficusal	−6.7
p-hydroxybenzaldehyde	−6.1
Vanillic acid	−6
Aeginetic acid	−5.8
Stigmasterol	−5.3
Beta-sitosterol	−5.1
Dehydrodiconiferyl alcohol gamma-O-beta-D-glucopyranoside	−5.1
Balanophonin	−4.9
2′-acetyl acteoside	4.8
Aeginetolide	−4.5
Dehydrodiconiferyl alcohol 4-O-beta-D-glucopyranoside	−3
Catechin hydrate	−1.8
Rutin hydrate	−1.8
Aegineoside	−1.3
Beta-sitosteryl glucoside	−0.8
Acteoside	−0.1
Cistanoside C	0.2

#### 3.2.2 Molecular dynamics simulation

Among the phytoconstituents, myricetin has the third highest concentration in the extract. However, the most important thing is that it forms hydrogen bonds with residues of hinge, linker, and DFG regions. Therefore, all atom molecular dynamics simulation was carried out for myricetin complexed with the active and inactive conformation of VEGFR2 for 25ns. After the completion of the simulation, various structural properties were analyzed.

**Fig. 2:**
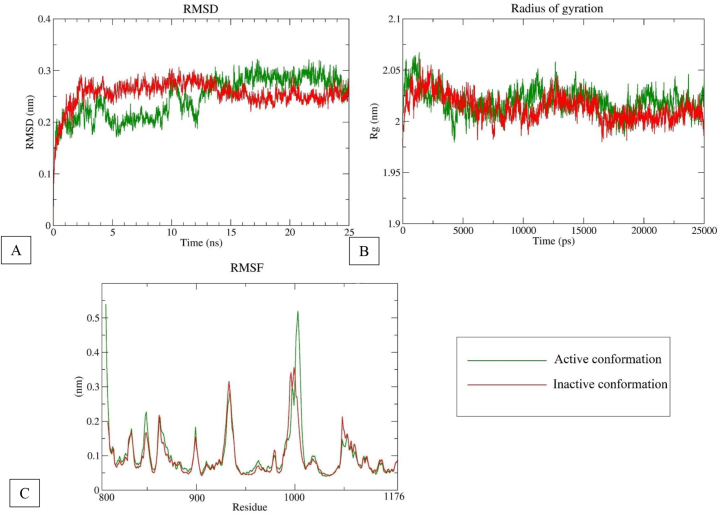
Molecular dynamics properties of DFG-in and DFG-out conformations of VEGFR-2 while bound with myricetin. (A) Root mean square deviation (RMSD) as a function of time, (B) Radius of gyration for backbone of VEGFR-2 and (C) Residual root mean square fluctuations (RMSF).

Root mean square deviation (RMSD) is the key property to justify a protein's structural stability and molecular integrity. In the present study, the conformational stability of active and inactive conformation of VEGFR2 were analyzed while complexed with myricetin. When myricetin binds, the inactive conformation of VEGFR2 shows little fluctuations, while the active conformation shows very consistency. In both cases, root mean square deviations were within 0.2 nm–0.3 nm during the course of dynamics (Fig. 2A). This indicates a very good stability of the conformations bound with ligand.

Radius of gyration (Rg) measures the compactness of protein structures during dynamics simulation. Rg was measured for myricetin bound active and inactive conformation of VEGFR2 at backbone of protein. During 25ns simulation, radius of gyration for both cases were within 0.05 nm with a very little fluctuation. Moreover, Rg of active and inactive conformations were almost overlapping (Fig. 2B). This indicates a good structural rigidness of protein.

Root mean square fluctuations (RMSF) measure the residual fluctuations to demonstrate structural flexibility. RMSF was calculated for active and inactive conformation of VEGFR2 for the 25ns of dynamics simulation (Fig. 2C). From RMSF plot, it is observed that both active and inactive conformations have a similar pattern of residual fluctuations. For some residues, there were more fluctuations for active conformation than for inactive conformation. This is due to more interaction with residues of active conformation and myricetin. Hinge region residues (916–919) fluctuations have not shown large deviation for both conformations. For DFG region residues (1046–1048), inactive conformation shows deviation, indicating more interaction with myricetin at the DFG region when the VEGFR2 is in an inactive conformation.

**Fig. 3:**
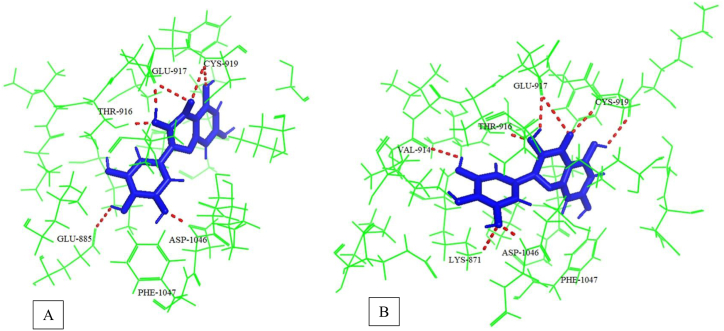
Snapshot of myricetin binding with VEGFR-2 at around 1ns of dynamics simulation. (A) hydrogen bonds with DFG-in conformation (B) hydrogen bonds with DFG-out conformation.

**Fig. 4:**
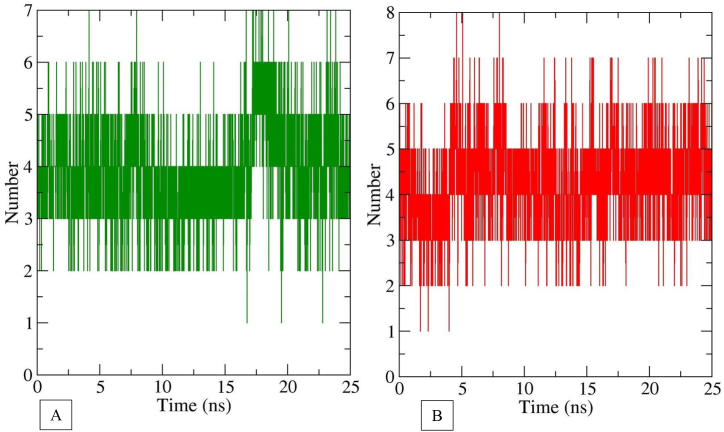
Number of hydrogen bonds formed between myricetin and VEGFR-2 at (A) DFG-in and (B) DFG-out conformation.

**Fig. 5:**
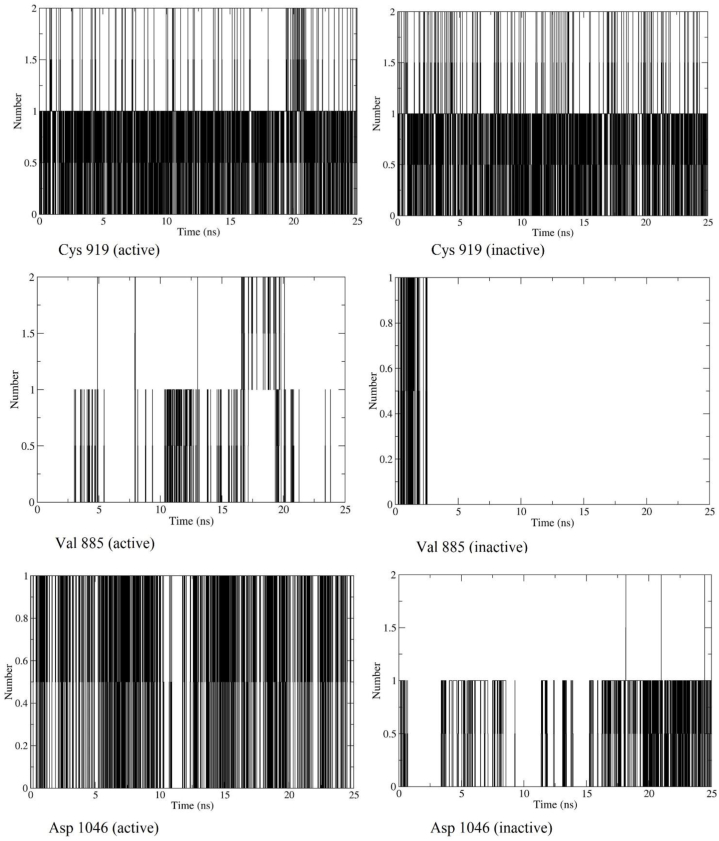
Hydrogen bonds between myricetin and key residues of VEGFR-2 in active and inactive conformation.

Fig. 3 shows the DFG-in and DFG-out conformation of VEGFR2 when docked with myricetin and possible hydrogen bonds formed between protein and ligand. The snapshots were taken at around 1 ns of trajectory files of both simulations. Interactive hydrogen bonds play a vast role in maintaining the stability of inhibitors and proteins. The number of hydrogen bonds formed during 25ns simulations was calculated for either case of VEGFR2 conformations. From Fig. 4, we have seen that myricetin forms mostly 3–5 hydrogen bonds when binds with VEGFR2 active conformation. When inactive conformation is considered, it is seen that 3–6 hydrogen bonds have been formed between ligand and protein. The number of hydrogen bonds is high enough to maintain the stability of myricetin and VEGFR2 complex in both conformations. In Fig. 5, it is observed that the Cys-919 residue of the hinge region consistently forms up to two hydrogen bonds with myricetin throughout the simulation in both conformations. Glu-885 linker residue forms hydrogen bonds (up to 2) with myricetin frequently in the active conformation, whereas inactive conformation does not form hydrogen bonds after 2.5ns. Asp-1046 of the DFG region forms more frequent hydrogen bonds with myricetin in active conformation than in inactive conformation.

## 4 Discussion

Phenolic compounds, which are widely present in plants, have garnered growing recognition for their possible involvement in the prevention and treatment of cancer. The antioxidant characteristics of these substances enable them to effectively counteract detrimental free radicals, therefore mitigating oxidative stress, which is a significant factor in the onset and progression of cancer. Additionally, these substances demonstrate anti-inflammatory characteristics, which have the potential to alleviate chronic inflammation, a well-established contributor to the onset of cancer. In addition, they exert modulation on diverse cellular signaling pathways, hence exerting influence on critical processes including cell proliferation, differentiation, and apoptosis, which play a crucial role in the progression of cancer. These chemicals have demonstrated the capacity to initiate programmed cell death (apoptosis) in cancer cells, impede the replication of cells, and potentially manifest antimetastatic properties, thus impeding the dissemination of cancer to distant anatomical sites. Moreover, these compounds have the potential to augment the immune system's ability to combat cancerous cells and exhibit a synergistic impact when combined with other therapeutic medicines, hence amplifying their efficacy. The aforementioned positive characteristics underscore the potential efficacy of phenolic substances in the management of cancer. The AiME was discovered to be significantly enriched with phenolic compounds, which may serve as a key source of anticancer medications.

Among the phytocompounds identified in the methanol extract of *Aegineta indica* by HPLC, myricetin showed a similar binding position compared to the standard sunitinib. Myricetin has a binding energy of −8.5 kcal/mol, forming 6 hydrogen bonds when docked in the catalytic site of VEGFR2. Molecular dynamics simulation has been carried out to verify the binding ability of myricetin with VEGFR2. Two significant conformations of VEGFR2, active and inactive, were used to perform the dynamics simulation. Both conformations of VEGFR2 showed structural stability on the basis of RMSD, RMSF, and radius of gyration study while bound with myricetin. Around 3–6 hydrogen bonds formed during the simulation study, indicating that the ligand myricetin's attachment with VEGFR2 was consistent. Type-I VEGFR2 inhibitors (e.g., sunitinib, approved for stromal tumor and advanced renal cell carcinoma) target ATP binding sites and form 1–3 hydrogen bonds with Cys-919, Glu-917 residues of hinge region [19]. Type II VEGFR2 inhibitors (e.g. sorafenib) target the linker region amino acid residues Glu-885 and Asp-1046 [10]. In this study, in both simulations, myricetin formed 1–2 hydrogen bonds with Cys-919 key residue of VEGFR2 hinge region. The extension of hydrogen bonds with other residues of the linker DFG region improves selectivity and physicochemical properties [9]. In DFG-in conformation, myricetin has consistent hydrogen bonds with Asp-1046 and frequent hydrogen bonds with Glu-885 residues. This indicates a strong binding of myricetin with VEGFR2. In DFG-out conformation, hydrogen bond with myricetin and Asp-1046 was quite frequent. But in case of Glu-885, the hydrogen bond did not form after 2.5 ns due to the displacement of Phe-1047 around 0.1 nm. However, the all-around hydrogen bonds with other residues were higher during the simulation which indicated a stable binding of myricetin with VEGFR2 and thus inhibiting it.

## 5 Conclusion

The current investigation unveiled that the methanol extract of *A. indica* exhibited a high concentration of several polyphenolic components. Molecular docking study was conducted to assess the interaction between phenolic compounds and previously isolated compounds with VEGFR2. The study successfully revealed five potential inhibitors of VEGFR2. In the molecular dynamic simulation, it was observed that myricetin exhibited notable inhibitory activities against VEGFR2 among the five compounds. The potential mechanism underlying the anticancer effect of myricetin may involve the inhibition of VEGFR2, leading to a subsequent inhibition of ICAM-1 expression.

## CRediT authorship contribution statement

**Marjanur Rahman Bhuiyan:** Investigation, Methodology, Writing – original draft. **Khondoker Shahin Ahmed:** Investigation, Methodology, Writing – original draft. **Md Sharif Reza:** Plant collection , Investigation. **Hemayet Hossain:** HPLC-DAD analysis, Validation and Formal Analysis. **Syed Mumtahin Mannan Siam:** Molecular docking, Molecular Dynamics Simulation, Visualization, Formal analysis. **Shahriar Nayan:** Molecular docking, Molecular Dynamics Simulation, Visualization, Formal analysis. **Sarah Jafrin:** Molecular docking, Molecular Dynamics Simulation, Visualization, Formal analysis. **Sadikur Rahman Shuvo:** Conceptualization, Supervision, Writing- Reviewing and Editing. **A.F.M.Shahid Ud Daula:** Conceptualization, Supervision, Writing- Reviewing and Editing.

## Data availability statement

Data will be made available upon request.

## Additional information

No additional information is available for this research paper.

## Funding statement

This research did not receive any funding from public, private or any non-government organizations.

## Declaration of competing interest

The authors declare that they have no known competing financial interests or personal relationships that could have appeared to influence the work reported in this paper.
