# Supplementary material for: Prediction of angiogenesis suppression by myricetin from Aeginetia indica via inhibiting VEGFR2 signaling pathway using computer-aided analysis
Source: Heliyon. 2025 Jan 7;11(2):e41749. doi: 10.1016/j.heliyon.2025.e41749 (PMC11786634; doi:10.1016/j.heliyon.2025.e41749)
Supplement: Multimedia component 1 [file mmc1.docx]

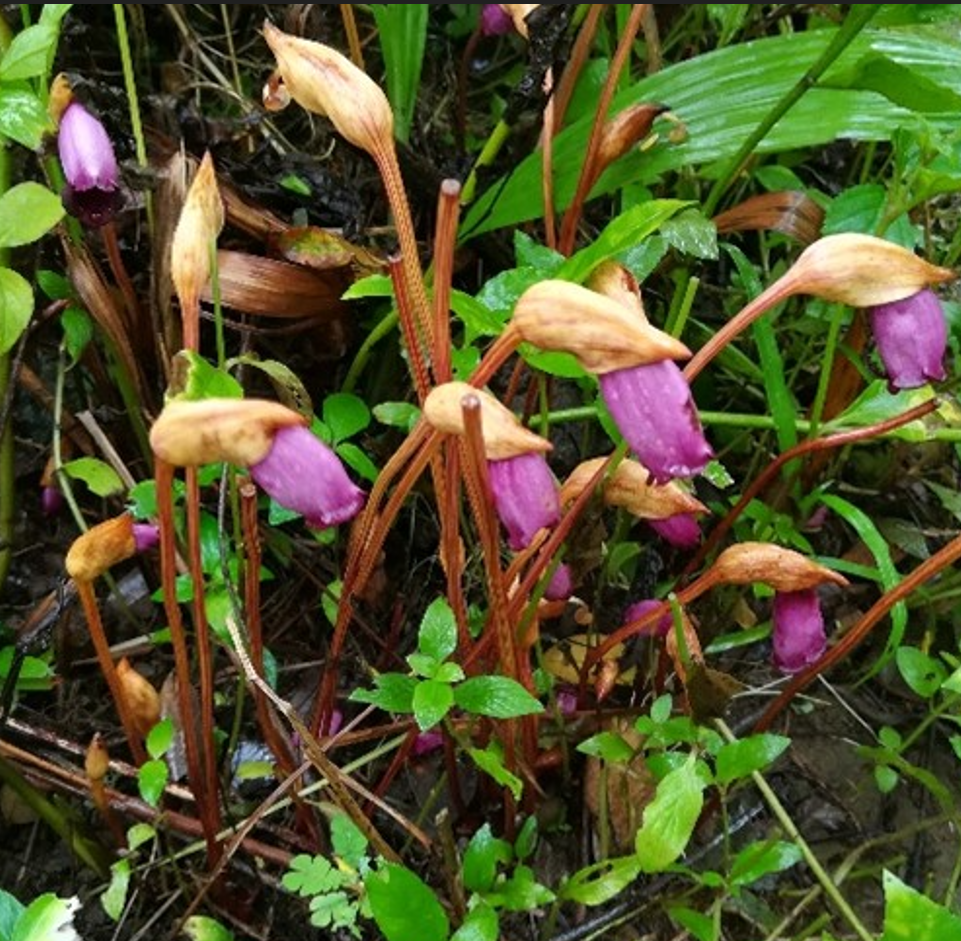


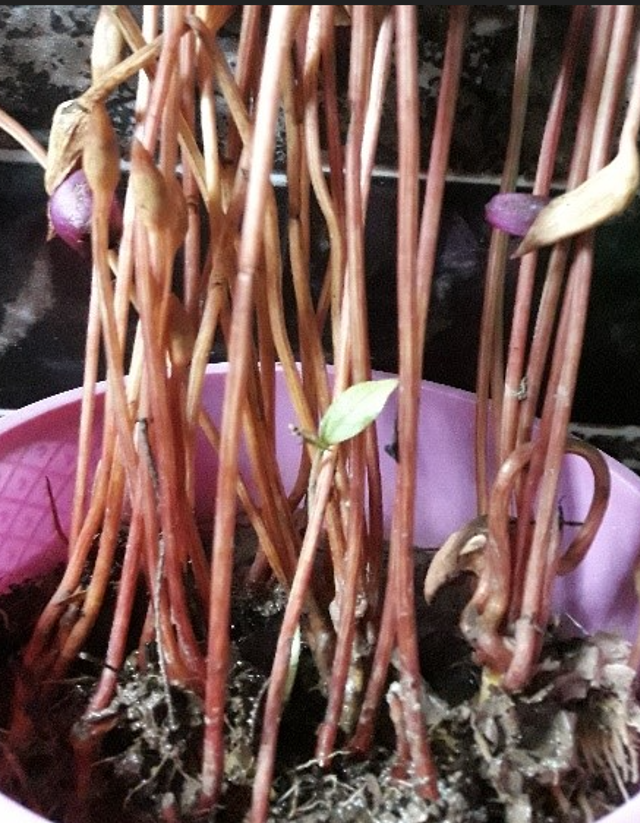

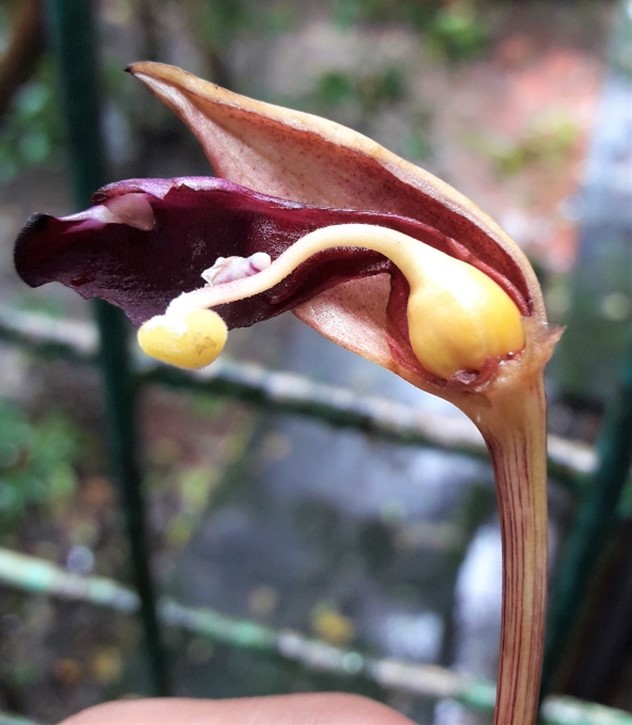


A

B

C

D

**Supplementary Figure 1:** *Aeginetia indica,* whole plant (A), stem (B), flower (C) and Ovary, Corolla, Calyx (D)

a)
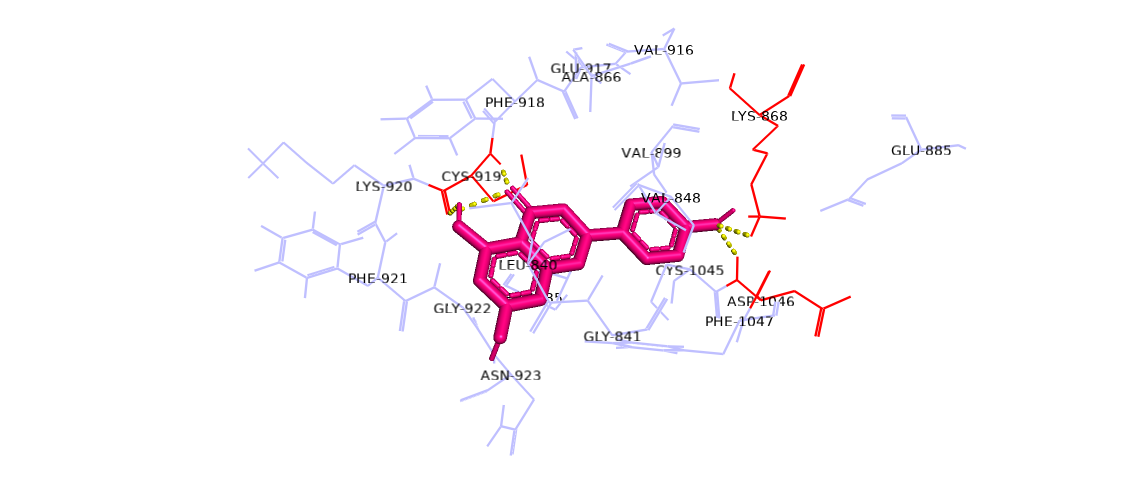
 b)
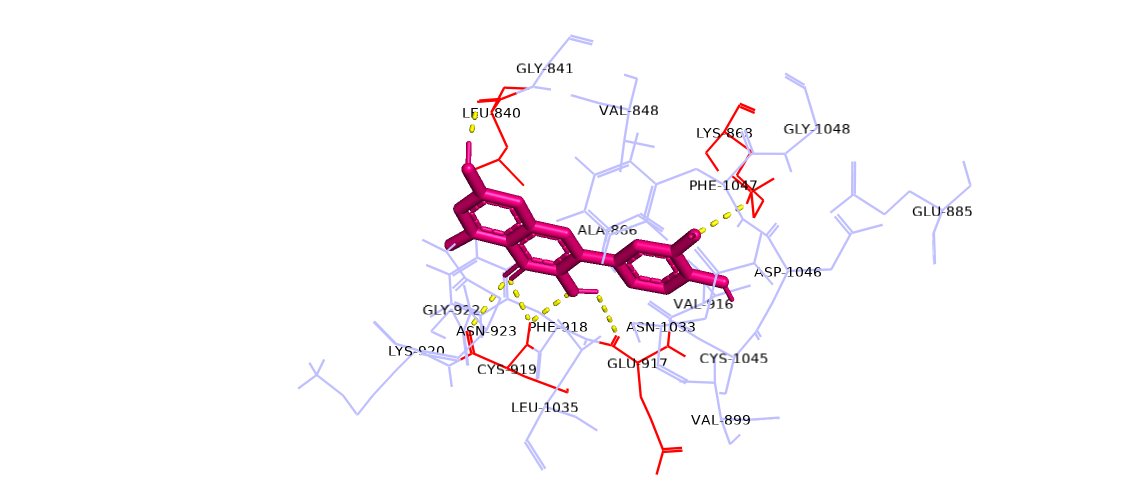
c)
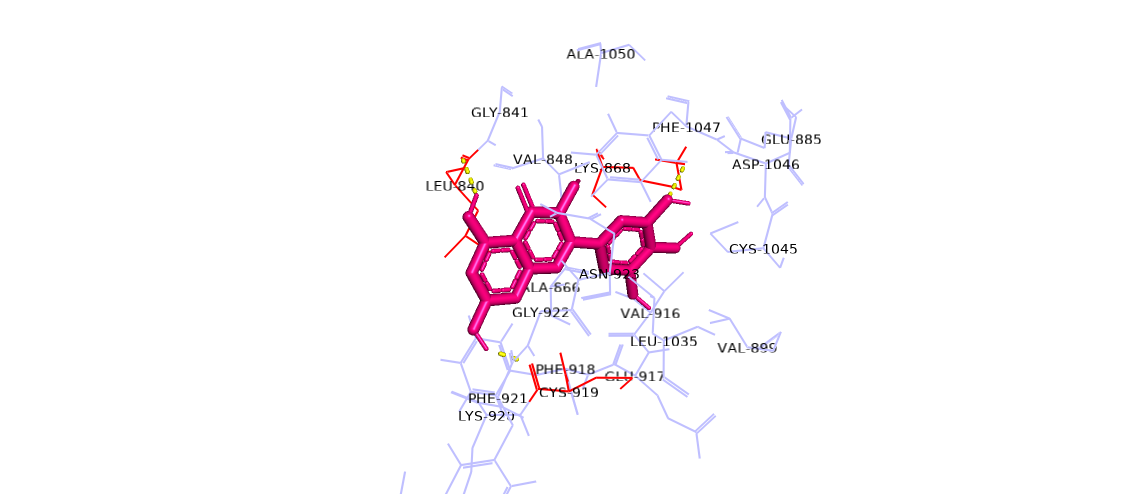


d)
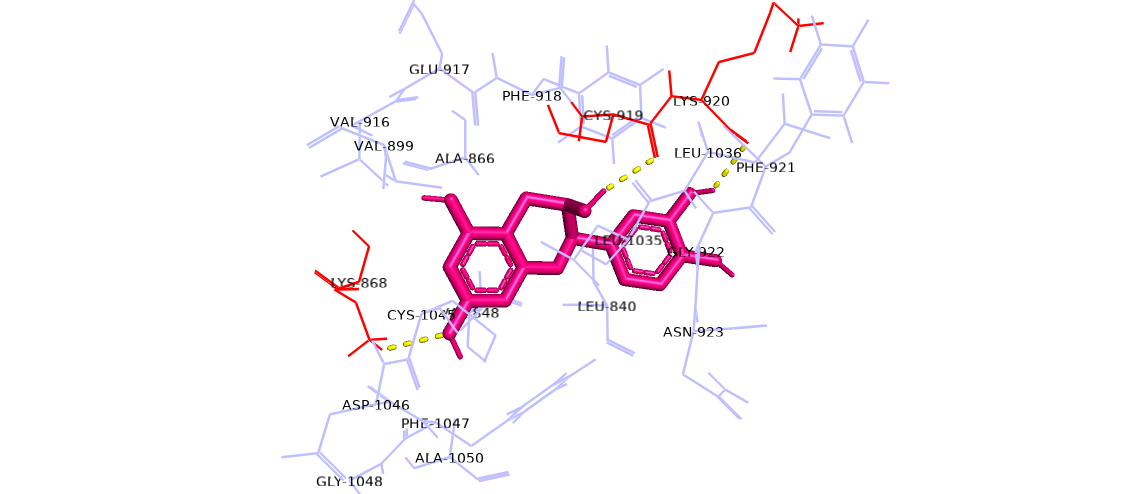

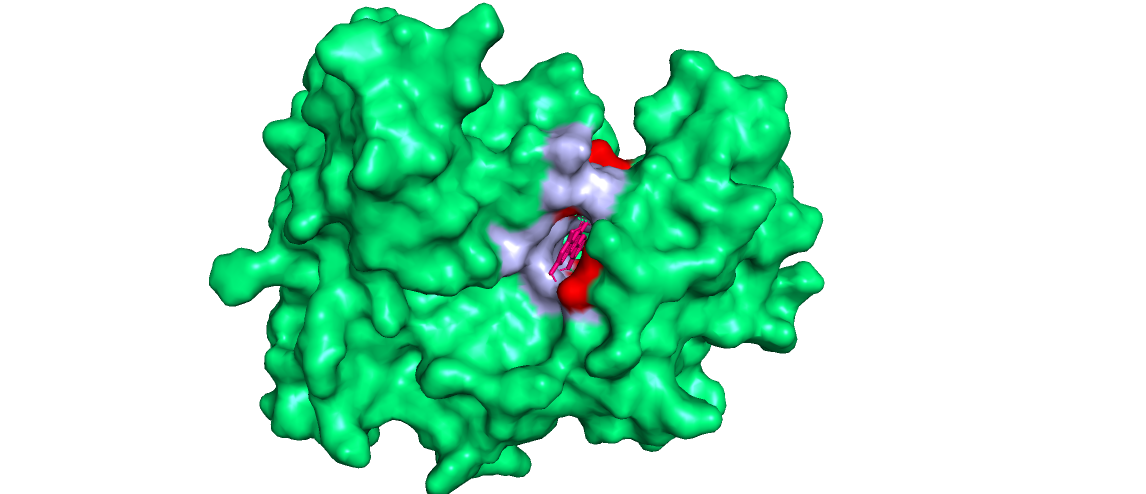
 e)
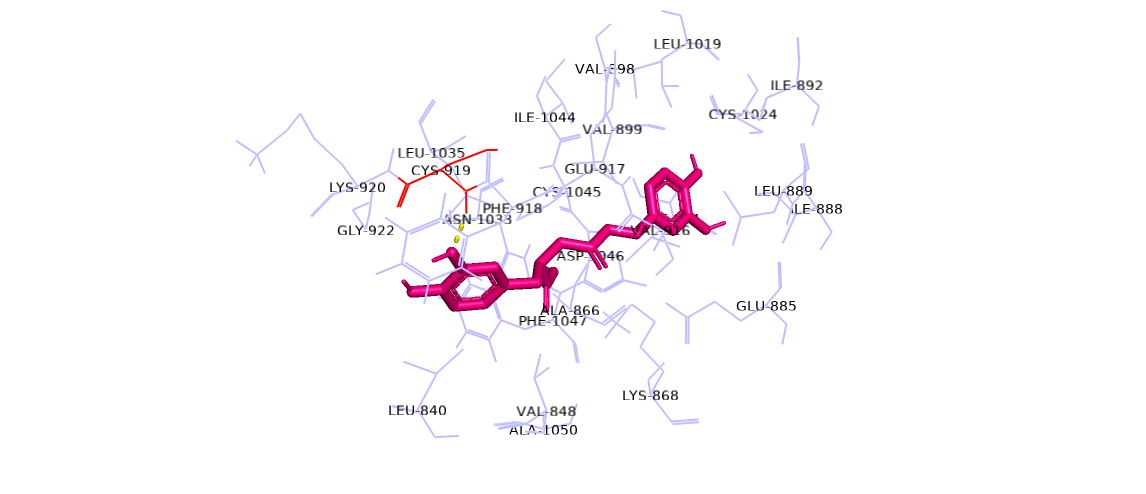


f)
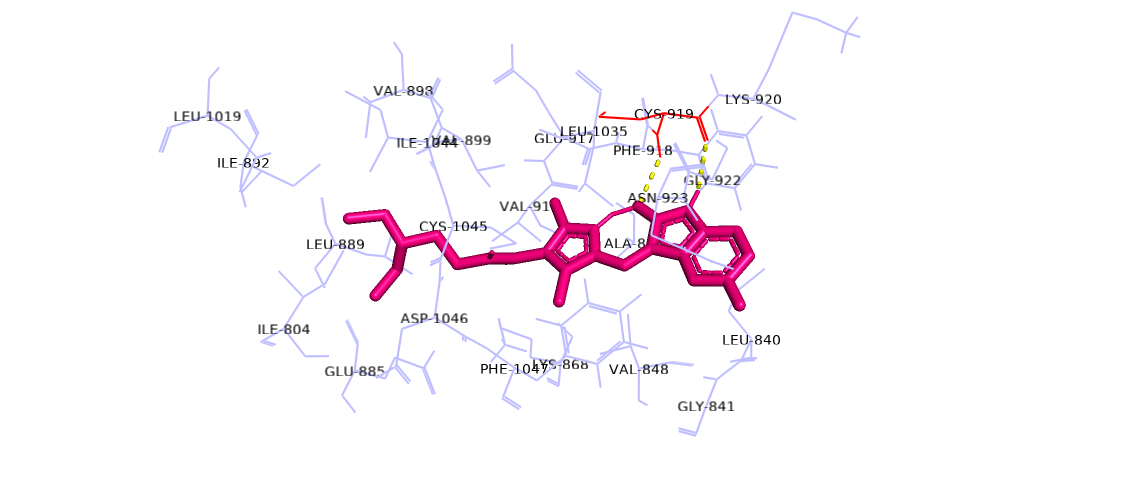
 g)
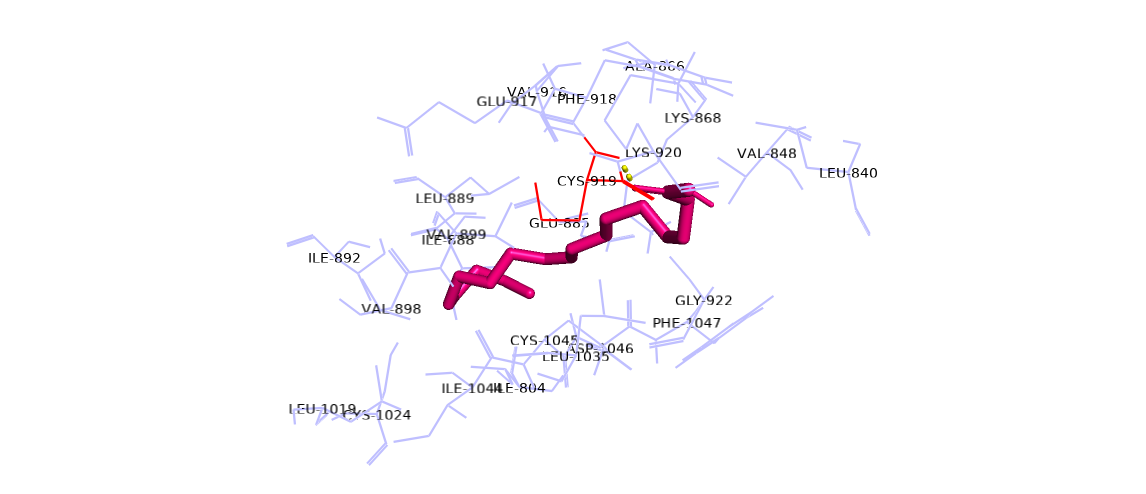


**Supplementary Figure 2:** Possible protein-ligand interactions of selected phytoconstituents from *Aeginetia indica* and standard sunitinib with VEGFR2 (PDB ID: 4AGD). Phytoconstituents are as follows: a) Apigenin, b) Quercetin, c) Myricetin, d) Epicatechin, e) Rosmarinic acid, f) Sunitinib, g) Oleic acid; Color indicators: Red: Amino acid residues forming hydrogen bonds with VEGFR2 protein; Yellow Dots: Hydrogen Bonds; Gray: Amino acid residues forming hydrophobic bonds with VEGFR2 protein; Pink: Ligand.

**Supplementary Table 1:** Origin and purity of phenolic compounds used in HPLC-DAD analysis.

| **Standard Compounds** | **Origin** | **Purity** |
| --- | --- | --- |
| Catechin hydrate | Sigma-Aldrich, Germany | ≥96% |
| (-) Epicatechin | Sigma-Aldrich, Germany | ≥90% |
| Caffeic acid | Sigma-Aldrich, Germany | 98% |
| Vanillic acid | Sigma-Aldrich, Germany | 97% |
| Rutin hydrate | Sigma-Aldrich, Germany | ≥94% |
| p-Coumaric acid | Sigma-Aldrich, Germany | ≥98% |
| Trans-Ferulic acid | Sigma-Aldrich, Germany | 99% |
| Rosmarinic acid | Sigma-Aldrich, Germany | ≥98% |
| Myricetin | Sigma-Aldrich, Germany | ≥96% |
| Quercetin | Sigma-Aldrich, Germany | ≥95% |
| Trans-Cinnamic acid | Sigma-Aldrich, Germany | ≥99% |

**Supplementary Table 2:** Relative standard deviations (RSD) obtained for each phenolic compound in AiME.

| **Standard Compounds** | **Peak area**  **(Mean ± %RSD)** | **Tailing factor (Mean)** | **Theoretical plate (Mean)** | **Rs** |
| --- | --- | --- | --- | --- |
| Catechin hydrate | 404451.50 ± 1.50 | 1.80 | 21313.57 | 1.915 |
| (-) Epicatechin | 1463.50 ± 1.40 | 0.97 | 60034.61 | 6.553 |
| Caffeic acid | 4056.00 ± 1.36 | 1.53 | 30527.52 | 2.051 |
| Vanillic acid | 10114.50 ± 1.81 | 1.63 | 28005.38 | 2.760 |
| Rutin hydrate | 1238.00 ± 1.60 | 1.02 | 160433.73 | 11.481 |
| p-Coumaric acid | 12228.50 ± 0.83 | 1.68 | 51743.78 | 2.606 |
| Trans-Ferulic acid | 4417.00 ± 1.76 | 1.44 | 62416.99 | 6.500 |
| Rosmarinic acid | 426983.50 ± 0.94 | 1.74 | 131513.12 | 9.243 |
| Myricetin | 6762.00 ± 1.84 | 1.27 | 127398.52 | 3.284 |
| Quercetin | 614605.00 ± 1.20 | 1.73 | 201721.12 | 15.049 |
| Trans-Cinnamic acid | 1993803.00 ± 1.45 | 1.82 | 256512.76 | 6.591 |
| **Acceptable limits** | **%RSD ≤ 2.0** | **≤ 2.0** | **˃2000** | **≥1.5** |

**Supplementary Table 3:** Figures of merit obtained for the analysis of phenolic compounds in AiME.

| **Standard Compounds** | **Retention time (minute)** | | ***R²** | ***LOD**  **(ppm)** | ***LOQ**  **(ppm)** | **Area** |
| --- | --- | --- | --- | --- | --- | --- |
|  | **Standard** | **Sample** |  |  |  |  |
| Catechin hydrate | 21.586 | 21.615 | 0.16 | 0.16 | 0.48 | 162717 |
| (-) Epicatechin | 24.770 | 24.680 | 0.15 | 0.15 | 0.44 | 390274 |
| Caffeic acid | 25.171 | 25.162 | 0.07 | 0.07 | 0.20 | 315795 |
| Vanillic acid | 25.451 | 25.468 | 0.09 | 0.09 | 0.28 | 631706 |
| Rutin hydrate | 29.913 | 30.047 | 0.07 | 0.07 | 0.23 | 5135049 |
| p-Coumaric acid | 31.053 | 30.955 | 0.06 | 0.06 | 0.19 | 19224 |
| Trans-Ferulic acid | 33.186 | 33.079 | 0.09 | 0.09 | 0.26 | 308264 |
| Rosmarinic acid | 36.563 | 36.423 | 0.16 | 0.16 | 0.49 | 398177 |
| Myricetin | 37.675 | 37.572 | 0.10 | 0.10 | 0.30 | 1042797 |
| Quercetin | 42.199 | 42.064 | 0.04 | 0.04 | 0.13 | 2305831 |
| Trans-Cinnamic acid | 44.742 | 44.641 | 0.05 | 0.05 | 0.15 | 150495 |

* Correlation Coefficient (R²); LOD, limit of detection; LOQ, limit of quantiﬁcation.

**Supplementary Table 4:** Bond Analysis among the best phytoconstituents and Sunitinib (Standard) with VEGF2 (PDB: 4AGD)

| Compound | Types of Interactions | | | |
| --- | --- | --- | --- | --- |
| Apigenin  (CID: 5280443) | **H Bonding Residue**  **(Distance)** | | **Total Hydrogen Bond** | **Hydrophobic Bond Residue (Type)** |
|  | **Conventional** | Cys919 (1.913)  Asp1046 (2.610)  Cys919 (2.655) | 3 | Leu840 (Pi-Alkyl)  Leu840 (Pi-Alkyl)  Val848 (Pi-Alkyl)  Ala866 (Pi-Alkyl)  Cys919 (Pi-Alkyl)  Leu1035 (Pi-Alkyl)  Val848 (Pi-Alkyl)  Val899 (Pi-Alkyl)  Leu1035 (Pi-Alkyl)  Cys1045 (Pi-Alkyl) |
| Quercetin  (CID: 5280343) | **Conventional** | Lys868 (2.492)  Cys919 (2.208)  Cys919 (2.300)  Glu917 (2.651)  Leu840 (1.988) | 5 | Leu840 (Pi-Sigma)  Phe1047 (Pi-Pi T-shaped)  Leu840 (Pi-Alkyl)  Val848 (Pi-Alkyl)  Ala866 (Pi-Alkyl)  Cys919 (Pi-Alkyl)  Leu1035 (Pi-Alkyl)  Val848 (Pi-Alkyl)  Ala866 (Pi-Alkyl)  Val899 (Pi-Alkyl)  Val916 (Pi-Alkyl)  Leu1035 (Pi-Alkyl)  Cys1045 (Pi-Alkyl) |
| Myricetin  (CID: 5281672) | **Conventional** | Glu917 (2.150)  Leu840 (2.223)  Cys919 (2.065) | 4 | Phe918 (Pi-Pi Stacked)  Phe1047 (Pi-Pi T-shaped)  Leu840 (Pi-Alkyl)  Leu840 (Pi-Alkyl)  Val848 (Pi-Alkyl)  Leu1035 (Pi-Alkyl)  Val848 (Pi-Alkyl)  Ala866 (Pi-Alkyl)  Val899 (Pi-Alkyl)  Leu1035 (Pi-Alkyl)  Cys1045 (Pi-Alkyl) |
|  | **Carbon** | Phe1047 (2.534) |  |  |
| Epicatechin  (CID: 72276) | **Conventional** | Lys868 (2.545)  Lys920 (2.302)  Cys919 (2.357) | 3 | Phe918 (Pi-Pi Stacked)  Phe1047 (Pi-Pi T-shaped)  Leu840 (Alkyl)  Val848 (Alkyl)  Ala866 (Alkyl)  Cys919 (Alkyl)  Leu1035 (Alkyl)  Val848 (Pi-Alkyl)  Ala866 (Pi-Alkyl)  Val899 (Pi-Alkyl)  Leu1035 (Pi-Alkyl)  Cys1045 (Pi-Alkyl)  Leu840 (Pi-Alkyl) |
| Rosmarinic Acid  (CID: 5281792) | **Conventional** | Lys868 (2.994)  Cys919 (1.966)  Cys919 (2.677) | 5 | Ile804 (Pi-Alkyl)  Leu889 (Pi-Alkyl)  Val899 (Pi-Alkyl)  Leu840 (Pi-Alkyl)  Val848 (Pi-Alkyl)  Ala866 (Pi-Alkyl)  Leu1035 (Pi-Alkyl) |
|  | **Carbon** | Lys868 (1.483)  Phe1047 (2.261) |  |  |
| Sunitinib  (CID: 5329102) | **Conventional** | Cys919 (2.391)  Cys919 (2.459) | 5 | Leu840 (Pi-Sigma)  Leu840 (Pi-Sigma)  Phe918 (Pi-Pi Stacked)  Ala866 (Alkyl)  Val848 (Alkyl)  Cys1045 (Alkyl)  Val899 (Alkyl)  Val916 (Alkyl)  Leu889 (Alkyl)  Ile804 (Alkyl)  Leu889 (Alkyl)  Val898 (Alkyl)  Val899 (Alkyl)  Phe1047 Pi-Alkyl  Val848 (Pi-Alkyl) (2)  Val866 (Pi-Alkyl) (2)  Val899 (Pi-Alkyl)  Val916 (Pi-Alkyl)  Cys919 (Pi-Alkyl) (2)  Leu1035 (Pi-Alkyl) (2) |
|  | **Carbon** | Lys868 (1.694)  Asp1046 (2.691)  Val899 (2.725) |  |  |
| Oleic Acid  (CID: 445639) | **Conventional** | Cys919 (2.076) | 2 | VAL848 (Alkyl)  ALA866 (Alkyl)  LYS868 (Alkyl)  VAL898 (Alkyl)  VAL899 (Alkyl) (3)  VAL916 (Alkyl) (2)  CYS1045 (Alkyl) (2)  LEU1035 (Alkyl)  LEU889 (Alkyl) (3)  ILE804 (Alkyl) (2)  ILE892 (Alkyl)  ILE888 (Alkyl)  PHE1047 (Alkyl) |
|  | **Carbon** | Phe918 (2.863) |  |  |
| P-Coumaric Acid  (CID: 637542) | **Conventional** | Lys868 (2.450)  Cys919 (2.606) | 4 | Phe918 (Pi-Pi Stacked)  Leu840 (Alkyl)  Val848 (Alkyl)  Ala866 (Alkyl)  Cys919 (Alkyl)  Leu1035 (Alkyl) |
|  | **Carbon** | Lys868 (2.804)  Phe1047 (1.952) |  |  |
| Trans-Cinnamic Acid  (CID: 444539) | **Conventional** | Lys868 (2.475) | 2 | Phe918 (Pi-Pi Stacked)  Leu840 (Pi-Alkyl)  Val848 (Pi-Alkyl)  Ala866 (Pi-Alkyl)  Cys919 (Pi-Alkyl)  Leu1035 (Pi-Alkyl) |
|  | **Carbon** | Phe1047 (1.992) |  |  |
| Trans-Ferulic Acid  (CID: 445858) | **Conventional** | Lys868 (2.454) | 3 | Phe918 (Pi-Pi Stacked)  Leu840 (Alkyl)  Phe1047 (Pi-Alkyl)  Leu840 (Pi-Alkyl)  Val848 (Pi-Alkyl)  Ala966 (Pi-Alkyl)  Cys919 (Pi-Alkyl)  Leu1035 (Pi-Alkyl) |
|  | **Carbon** | Lys868 (2.827)  Phe1047 (1.961) |  |  |
| Caffeic Acid  (CID: 689043) | **Conventional** | Cys919 (2.055)  Glu917 (2.274) | 3 | Val848 (Pi-Sigma)  Phe1047 (Pi-Pi T-shaped)  Ala866 (Pi-Alkyl)  Leu1035 (Pi-Alkyl)  Cys1045 (Pi-Alkyl) |
|  | **Carbon** | Lys868 (2.606) |  |  |
